# Landscape connectivity among coastal giant salamander (*Dicamptodon tenebrosus*) populations shows no association with land use, fire frequency, or river drainage but exhibits genetic signatures of potential conservation concern

**DOI:** 10.1371/journal.pone.0268882

**Published:** 2022-06-08

**Authors:** Giorgia G. Auteri, M. Raquel Marchán-Rivadeneira, Deanna H. Olson, L. Lacey Knowles

**Affiliations:** 1 Department of Ecology and Evolutionary Biology, University of Michigan, Ann Arbor, Michigan, United States of America; 2 Edison Biotechnology Institute, Ohio University, Athens, Ohio, United States of America; 3 Pacific Northwest Research Station, United States Department of Agriculture, Forest Service, Corvallis, Oregon, United States of America; Universita degli Studi della Tuscia, ITALY

## Abstract

Determining the genetic consequences of both historical and contemporary events can clarify the effects of the environment on population connectivity and inform conservation decisions. Historical events (like glaciations) and contemporary factors (like logging) can disrupt gene flow between populations. This is especially true among species with specialized ecological requirements and low dispersal ability, like amphibians. We test for the genetic consequences of historical and contemporary disturbances in the coastal giant salamander (*Dicamptodon tenebrosus*) in the Pacific Northwest of the United States. We consider predictions based on the contemporary landscape (habitat connectivity, logging, forest fires, and topography), in addition to relatively ancient post-Pleistocene range expansion (following the last glacial retreat). To assess local versus larger-scale effects, we sampled 318 individuals across 23 sites, which were clustered in five sampling regions. Genetic variation was assessed using five microsatellite markers. We found evidence of (i) historical regional isolation, with decreased genetic diversity among more recently colonized northern sites, as well as (ii) high levels of inbreeding and loss of heterozygosity at local scales, despite relatively low overall population differentiation (*F*_*ST*_) or strong evidence for population bottlenecks. Genetic diversity was not associated with contemporary disturbances (logging or fire), and there were no detectable effects on the genetic connectivity of populations based on intervening landscape features (habitat fragmentation and topography). However, lower genetic diversity in more northern regions indicates a lag in recovery of genetic diversity following post-Pleistocene expansion. Additionally, some populations had evidence of having undergone a recent genetic bottleneck or had high inbreeding (*F*_*IS*_) values. Lower genetic diversity in more northern sites means populations may be more vulnerable to future environmental changes, and managing for connectivity alone may not be sufficient given low mobility. Recent apparent reductions in some populations were not clearly linked to anthropogenic disturbances we examined. This suggests the type of disturbances this species is sensitive to may not be well understood.

## Introduction

Both contemporary and historical factors can contribute to the geographic distribution of genetic variation. These factors can increase the relative isolation of individuals, consequently reducing gene flow, population abundance, and genetic diversity. Considering genetic signatures across landscapes can help link causes to consequences, and further guide conservation efforts by identifying populations with low genetic diversity that have recently experienced habitat disturbances. Genetic connectivity and diversity can be precursors for sensitivity to demographic changes, disease events, or future climatic changes (e.g., [1–4]). If gene flow becomes disrupted by contemporary changes to landscapes [5–7], mitigation might include restoring the focal habitats or the surrounding matrix in fragmented landscapes (e.g. [8–11]). Disruptions to gene flow may also have historical signatures, such as when populations become isolated as a consequence of shifting species distributions driven by glaciations [12–14]. This historical isolation might impose similar conservation vulnerabilities as those from human-induced fragmentation. However, the management of these populations would call for different strategies (see [15, 16]). As such, both historical and contemporary factors may need to be considered when developing management and conservation plans (see [15, 17]), except when one factor is dominant [18, 19].

The degree to which contemporary and historical factors contribute to population isolation will vary across taxonomic and geographic scales. Historical and contemporary factors may be important for species that have persisted or expanded into areas that have undergone pronounced climatic shifts, such as previously glaciated areas. Species with limited dispersal and specialized habitat associations may be particularly susceptible to the negative consequences of reduced connectivity.

Amphibians are often reliant on spatially patchy and ephemeral habitats (e.g., seasonal pools for early development), and have high habitat specificity and strict ecophysiological requirements [20, 21]. Their dependency on multiple habitats means that disturbance events to any one of the habitats can have devastating effects on populations (e.g., [22]). Interconnectivity between habitats required for life-history functions (e.g., breeding, foraging, and overwintering) [23–25] is essential for population persistence [21, 26, 27]. Over larger geographic scales, interconnectivity among populations may also be critical for population persistence. This is especially true when metapopulation dynamics are at play. For example, when populations have been displaced to multiple refugia and must track shifting habitat conditions across large geographic scales [14, 28, 29], or if breeding sites are located in isolated microhabitats such as pools [30] but post-breeding life functions (such as foraging and dispersal) occur in differing habitats. To date, 41% of amphibian species are of conservation concern [31], and management efforts increasingly focus on facilitating species movements among required habitat types (within populations) and across geographic scales (among populations).

Here, we test hypotheses about the effects of contemporary and historical factors on within- and among-population connectivity in the coastal giant salamander (*Dicamptodon tenebrosus*) from the Pacific Northwest (PNW) of the United States. The species breeds in naturally fragmented and disturbance-prone headwater habitats [22, 26, 27]. Headwater stream-associated amphibians often use streams for breeding and larval development, but transform to terrestrial forms and use riparian and upland forest habitats for foraging, overwintering, and dispersal (e.g., [22]). The coastal giant salamander is associated with moist forests and has limited dispersal ability [32–34]. Potential barriers and disturbances in the system could occur in either terrestrial or aquatic habitats. For example, headwaters are initiation points for downslope debris torrents [35, 36], and terrestrial habitat in the PNW has been fragmented by timber harvest, agriculture, and rural community development (e.g., [36–38]). Natural barriers to gene flow may include ridgelines (e.g., that define different stream basins) [39] and unsuitable downstream aquatic habitats that are too warm, too large, and possibly include predators (e.g., [40, 41]). Moreover, the PNW has been subject to climatic changes associated with the Pleistocene glacial cycles. Multiple isolated refugia have been proposed within the PNW, including the mesic forests of the Oregon Coast Range and Cascade Range [42].

Several studies have addressed the responses of PNW stream-associated salamanders to timber harvest practices. For example, In British Columbia, Canada, mobility of coastal giant salamanders was similar at sites where streamside riparian buffers were left intact during upland logging compared to fully forested sites, whereas logging these buffers significantly reduced salamanders’ movements [43]. In western Oregon, USA, wider buffers with upland thinning were associated with higher densities of coastal giant salamanders [44]. Logging of buffers was also associated with decreased genetic diversity in the species [34]. Maintaining buffers is therefore likely critical to mitigating both adverse effects on movements and abundance in this species. This is corroborated by the sensitivity of population connectivity in two other salamander species in the region (*Rhyacotriton* spp.) to land-cover type and the presence of roads [45]. However, there are ecological differences between coastal giant salamanders and *Rhyacotriton* spp. hypothesized to result in varying vulnerabilities to disturbance (e.g., [46, 47]). This makes it unclear if similar management strategies would be effective across species. Additionally, both *Rhyacotriton* spp. are rarer than coastal giant salamanders, suggesting correspondingly lower resilience to disturbance and barriers. With the unique assemblages of amphibians and arthropods associated with headwaters in the PNW [48–51], understanding the species-specific factors affecting connectivity provides essential knowledge for maintaining long-term stability in these communities.

We test hypotheses based on the population genetic variation of the coastal giant salamander to evaluate the potential effects of contemporary and historical factors on species vulnerability. We focus our sampling on the Oregon Coast Range within the PNW. By sampling at both regional and local (i.e., within-region) scales, we were able to test both historical (geographically broader) and contemporary (more localized) factors. Specifically, we explore genetic diversity and connectivity as related to land-use, logging, fire, as well across different river drainages.

## Methods

### Sampling

**Fig 1:**
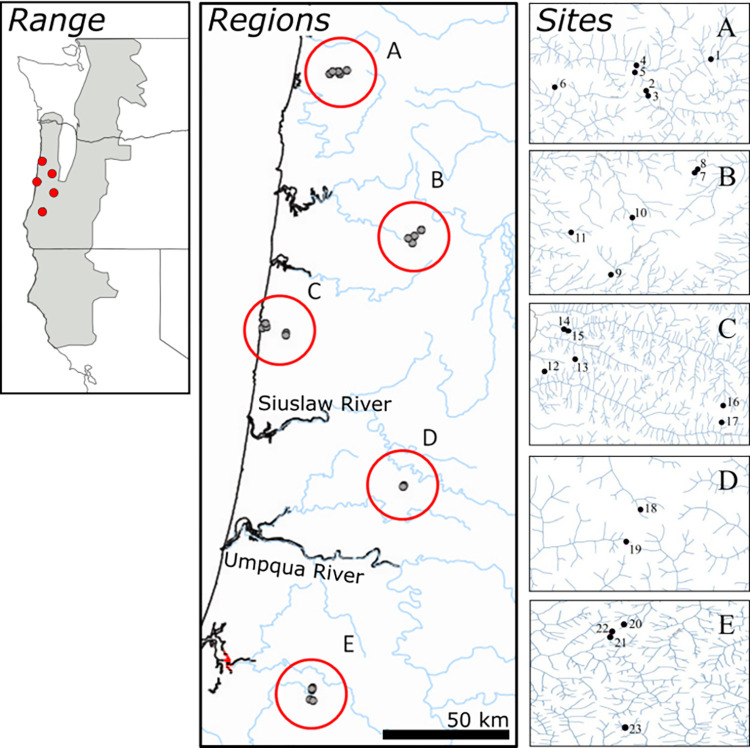
Sampling localities. The geographic range of coastal giant salamanders (left panel) showing the five sampled regions (center panel: circles A–E) along the coast of Oregon, USA, and details of sampled sites (labeled from 1 to 23; right panel; modified from Marchán-Rivadeneira, 2015 [52]). The left panel features species range information created using the IUCN Red List of Threatened Species [53] and other maps feature waterways from United States Geological Survey [54].

Tail or toe clips were collected from individual coastal giant salamanders across 23 headwater sites (designated 1–23) from five broadly separated regions (regions A–E; Fig 1) within the Oregon Coast Range. Sites were chosen to include either (i) streams in adjacent headwater drainages of distinct watersheds, such that freshwater habitat connectivity did not occur between them, or (ii) sites with interconnecting freshwater (tributary junctions downstream; Fig 1). Some regions included sampled streams in three distinct watersheds that met at their headwaters, providing three potential routes of terrestrial connectivity (i.e., “triads”) [27]. Within a region, the distance between sites varied from 0.12 to 7.01 km (mean = 2.79), with six sites sampled in regions A and C, five sites in region B, four sites in region E, but only two sites in region D (because of limited success in collecting animals). Region D was excluded from some analyses because of limited sampling (see details below). Tissues from a total of 318 individuals were collected, with between 4 and 29 individuals sampled per site (S1 Table), occurring during July through September 2010. Animal handling and samples followed ethics guidelines approved by the University of Michigan’s University Committee on Use and Care of Animals (UCUCA; permit #10233) and were collected under Oregon Scientific Permit #014–10.

### DNA extraction and genotyping

Tissue samples were stored in separate vials of 75% ethanol (EtOH) upon collection. DNEasy Tissue kits (Qiagen, Inc., Germantown, MD, USA) were used for DNA extractions, following manufacturer guidelines. Individuals were genotyped for five microsatellite loci (S1 Table) developed for this species using primers and protocols developed by Steele et al. (markers D6, D14, D17) [55] and Curtis and Taylor (Dte 11, Dte 14) [56].

### Quantification of molecular data

PCR products were genotyped, and alleles were manually scored using GeneMarker software (v. 1.97 SoftGenetics, State College, PA, USA). Potential genotyping errors, including null alleles, scoring errors, and allele dropout, were assessed using Micro-Checker (v. 2.3.3) [57]. Tests for significant deviations from Hardy-Weinberg equilibrium (HWE) and linkage disequilibrium (LD), as well as null allele frequency (*r*), were conducted for each locus using GenePop (v. 4.1) [58]; significant departures from HWE and LD were tested with 100 batches of 1000 iterations. Of the 318 individuals sampled, most (240) had data for all five markers, while 65 were missing data for one marker, 11 were missing data for 2, and two individuals only had information for one marker. Missing data for each marker ranged from 2% (Dte 11) to 13% (Dte 14), with other markers missing 3% (D6), 5% (D14), and 7% (D17). Allelic richness, observed heterozygosity (*H*_*o*_), expected heterozygosity (*H*_*e*_), and genetic differentiation between populations (*F*_*ST*_) were calculated using Fstat (v. 2.9.3) [59].

### Tests of genetic structure across space and drainages

Geographic structuring of genetic variation was evaluated in two complementary approaches: by principal component analysis (PCA) and a Structure analysis (v. 2.3.4) [60, 61]. The PCA was performed in R [62] using RStudio (v. 1.1.453) [63] using the *prcomp* function and visualized using the package ggplot2 [64]. Missing values were replaced with the per-locus mean. In the Structure analyses, *K-*values of 1 to 24 were run in the global analysis (i.e., all sample sites) without conditioning on geographic sites, using the admixture model with a 50,000 burn-in, followed by 500,000 repetitions. Ten independent runs were conducted for each *K*-value. We also conducted additional Structure analyses using *a priori* assignments. For these analyses, we conditioned by (a) population (*K* = 1–24), (b) drainage (*K* = 1–13), and (c) region (*K* = 1–6). The most likely *K*-value was estimated using Structure Harvester [65], and assignment probabilities of individuals’ ancestries were determined using Clumpak [66, 67].

We also evaluated isolation by distance (IBD) based on *F*_*ST*_ versus the minimum distances between sites using RStudio [62, 63] and ggplot2 [64]. We evaluated the relative contribution of IBD within and among regions. The significance of IBD was evaluated for the entire dataset and the subsets of the data for each within-region comparison using Monte-Carlo simulations performed in the R package adegenet [68] using 999 random replicates per test.

We examined the structuring of genetic variation as a function of watershed and drainage (S2 Table), controlling for the effects of geographic distance by analyzing the residuals of a linear model of between-site *F*_*ST*_ -values versus geographic distance, then testing whether these residuals were explained by boundaries between drainage basins. Specifically, differences among residuals based on drainage association (i.e., same versus different drainage membership) were analyzed using a Kruskal-Wallis test [69] with a Bonferroni correction [70, 71] for multiple comparisons (i.e., an alpha-threshold of 0.01 for significance). These tests were carried out using multiple watershed categorizations to accommodate uncertainty regarding relevant barriers for *D*. *tenebrosus* (explained below). The different categories for water catchments included: Hydrological Units (HUs) at three levels (HU 08, 10, and 12) [72], drainage basin (a continuous river system and its catchment area), and river systems separated by major waterways (“Level 1” streams) [73]. Larger waterways can act as barriers to headwater-associated amphibians due to increased currents, depths, temperatures, and predators (e.g., fish) [74, 75]. However, adult coastal giant salamanders have been found in larger rivers [76]. Applying different watershed scales (i.e., HU 8, 10, 12, complete drainages, or watersheds separated by Level 1 streams), the number of different watersheds represented by our sampled sites are either 7, 9, 13, 8, or 16, respectively (see S2 Table).

### Landscape effects on connectivity

The extent of different landcover classes was obtained from the National Land Cover Database (NLCD) from 2006 [77], given that the salamanders were sampled in 2010. We focused on terrestrial landcover types likely to impact this species. All sites at which we found salamanders were within the evergreen forest (NLDC category 42), suggesting that the proportion of evergreen forest between sites would facilitate terrestrial movements (i.e., evergreen forest would be negatively correlated with genetic differentiation, as measured by *F*_*ST*_). Conversely, habitat fragmentation and less desirable land cover types would increase genetic differentiation among sampling sites.

To quantify the landcover effects of areas separating sampled sites, we identified regions representing the shortest distances between each pair of sites in a region and created 80-m wide rectangles between the two sample sites (denoting a potential dispersal corridor). The width of rectangles (i.e., potential corridors) was chosen by quadrupling the maximum observed movement distance (of 20 m) [43] to hedge uncertainty in known mobility and edge effects. Each overlaid polygon was used to calculate the proportion and patchiness (Patch Cohesion Index) of the underlying landcover types using the Landscape Ecology plugin LECOS (v. 3.0) [78] in QGIS (v. 3.6.1) [79]. Although nine different landcover types occurred within the potential immigration corridors, we restricted downstream analyses to quantification based on two categories (Evergreen Forest and Shrub/Scrub), which were the two most common land-cover categories. To tests for the effects of land-cover, we fit a linear model to the *F*_*ST*_ *-*values and five different predictors: distance, proportion of evergreen forest, proportion of shrub/scrub, and patch cohesion of evergreen forest between sites, as well as whether sites were in the same watershed. We performed analyses in RStudio [62, 63]. Only a single watershed membership was included in this model, as determined by population differentiation discussed in the previous section. The best model was identified using AIC score and a backward stepwise regression model [80, 81].

### Influence of disturbance events on genetic diversity

We tested for the potential effects of disturbance events on genetic diversity using genetic bottleneck-tests within sites at each site using BOTTLENECK (1.2.02) [82]. This approach evaluates whether expected heterozygosity (*H*_*e*_) is significantly higher than the equilibrium heterozygosity (*H*_*eq*_). Significance was evaluated using a one-sided Wilcoxon sign rank test based on 1,000 iterations. Note that bottlenecks in this test are defined as severe population contractions resulting in a reduction of effective population size, not as a population contraction followed by an expansion. We used the developer’s recommended settings for microsatellite data for our loci and individual sample size. Specifically, the settings were a two-phase mutation model with 95% of single mutations, a multi-step variance of 12, and the mode shift turned off. In addition, based on program guidelines and subsequent methodological studies [82, 83], we restricted this analysis to sites for which at least 10 individuals had information from all five microsatellite loci (i.e. for a total of 14 sites, see S1 Table). We note that despite our exclusion of sites with limited sampling, the tests may still have reduced efficacy.

We also tested for correlations between genetic diversity (average allelic richness) and whether a disturbance event had occurred in the last 70 years via Kruskal-Wallis tests [69]. We compiled information on whether logging or fires had occurred in the past 70 years at each site from historical records and databases of wildfires that occurred (through federal sources; Bureau of Indian Affairs, Bureau of Land Management, Fish and Wildlife Service, National Park Service, and Forest Service, [84]; and historical satellite imagery on Google Maps, ending in 2016). We also noted whether trees had been maintained along the river during land-use disturbance events (in the case of fires, it was assumed that no riparian forest buffer remained).

## Results

All microsatellite loci were polymorphic. There were no consistent patterns of deviation from HWE, null alleles, or linkage disequilibrium across sites or across microsatellites, with the exception of the D6 locus (which was out of HWE). Consequently, all analyses were performed with and without the D6 locus, and because results were qualitatively similar, analyses using all loci are presented here (see S1 Text for results based on analyses with D6 excluded).

Genetic diversity was generally high (globally across all alleles 18–40 alleles per site, mean = 28.6; see S1 and S3 Tables). Per locus and geographic sampled site, the number of alleles (NA) ranged from 2–15, expected heterozygosity (*H*_*e*_) ranged from 0.07 to 1.00, and observed heterozygosity (*H*_*o*_) ranged from 0.00 to 1.00. The *F*_*IS*_-index per-population (averaged across loci) had a mean of 0.37 (SD ± 0.096) and ranged from 0.14 (site 11) to 0.57 (site 9; S3 Table). Per locus, *F*_*IS*_ ranged from -0.46 to 1.00 (S1 Table). Pairwise genetic differentiation between sampled sites ranged from an *F*_*ST*_ of 0.0 to 0.25 (maximum potential value is 1.00).

### Tests of genetic structure across space and drainages

**Fig 2:**
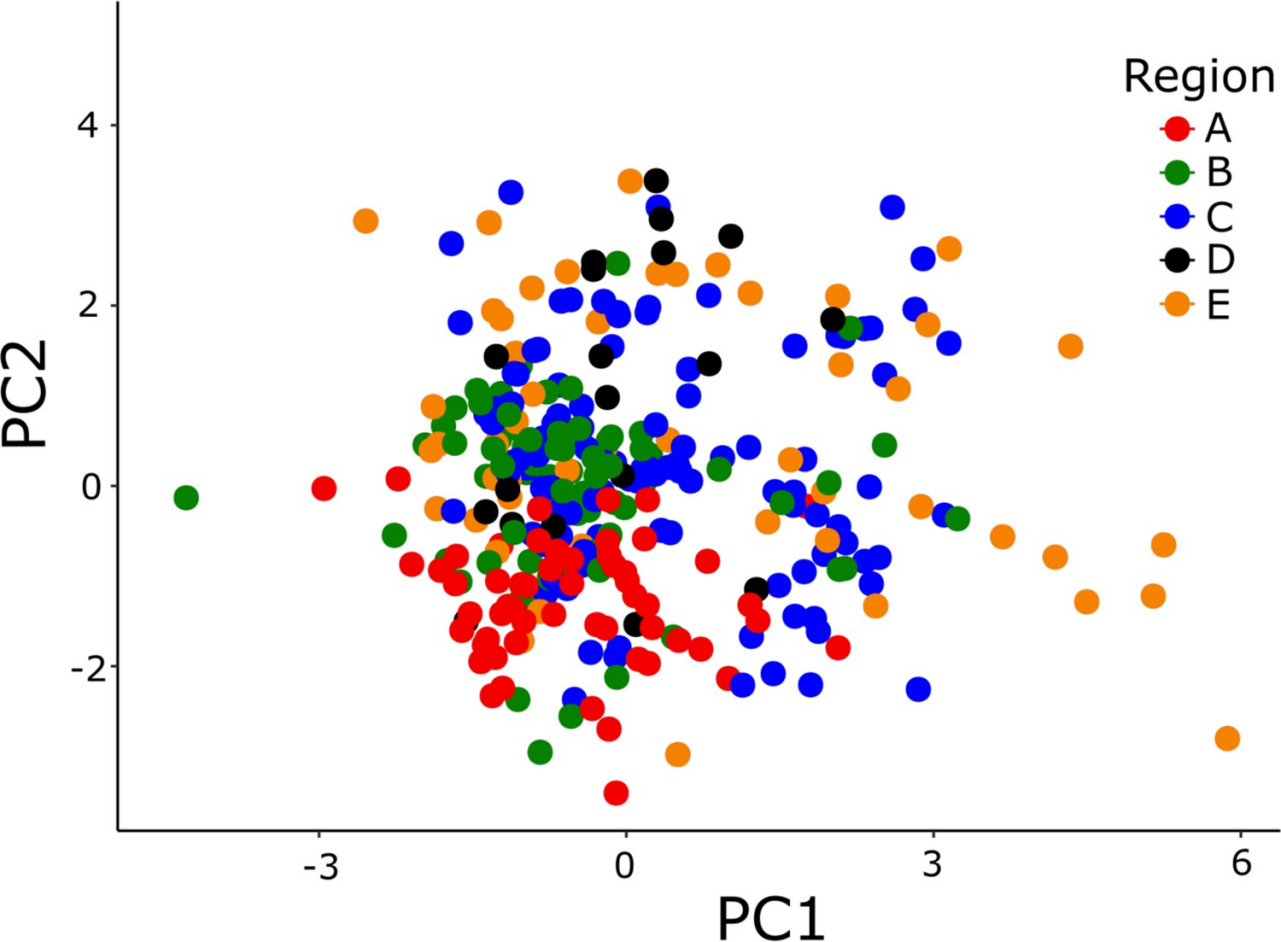
Principal component analysis of sampled individuals. The 318 individuals are color-coded by region (see Fig 1), where the first and second axes explained 21% and 18% of the variance, respectively.

**Fig 3:**
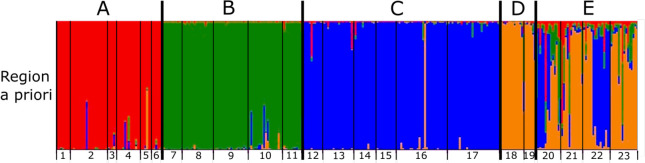
Plot of genetic STRUCTURE results. Individuals (vertical bars) are grouped by site (1–23) and region (A–E; separated by bold vertical lines; see Fig 1). Results are presented for the most likely number of genetic clusters, *K* = 4 when conditioning on region (see S2 Fig for runs with alternative priors).

The PCA showed overlap of some, but not all regions; individuals from the north (region A) did not overlap with individuals from the southern region (region E) on PC2. The northern region (A) also tended towards less genetic variation (i.e., occupied a smaller space in the PCA; Fig 2). Structure results generally supported regional differentiation, with a *K* = 4 as most probable (Fig 3), regardless of a-priori information used to condition assignments (i.e., conditioning on region, HU 8 watershed, or population; see S1 Fig). The one exception was for conditioning on sample site, which resulted in a most likely *K* = 3.

**Fig 4:**
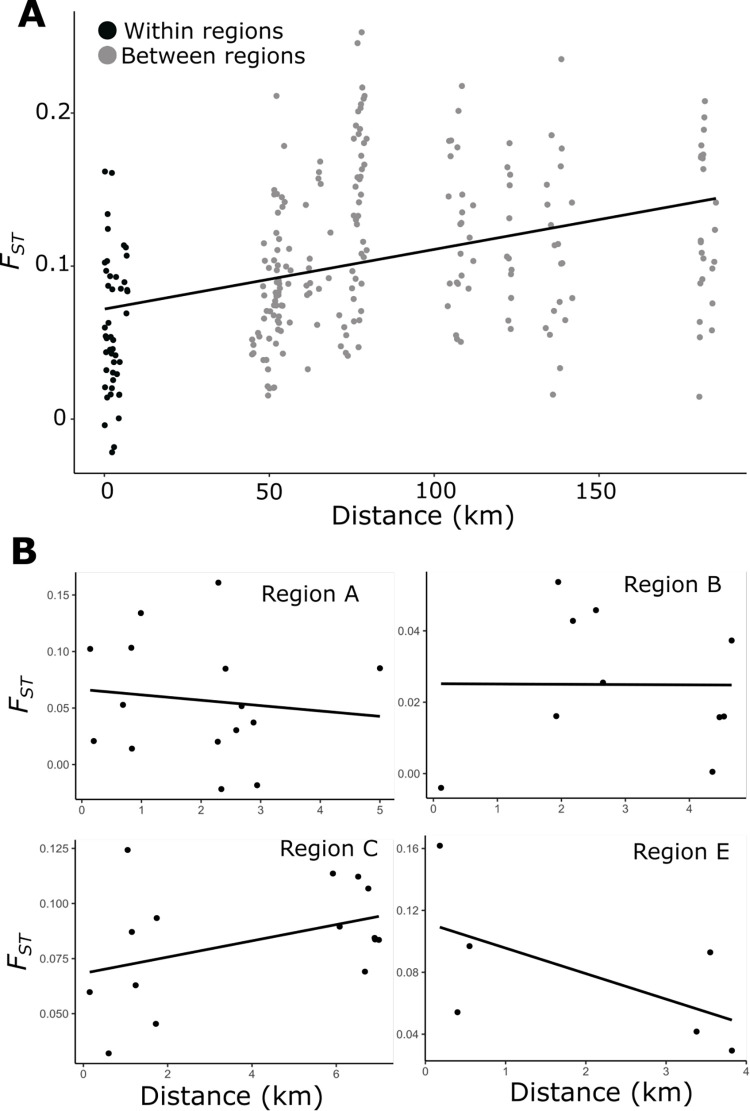
Isolation by distance plots. Segregation of allele frequencies (measured by *F*_*ST*_) is shown between pairs of sites versus geographic distance. A. All site pairs in this study, with comparisons within regions shown in black and comparisons between regions in grey. B. Within-region site comparisons, grouped by region (region D is omitted since only two sites were sampled).

IBD was significant across regions (*P* ≤ 0.001), but not within regions (*P* = 0.454). Within regions, there is pronounced variation in the levels of genetic differentiation across sites (Fig 4). Within-regions, drainage basins did not generally contribute significantly to observed genetic differentiation among sites. However, one of the five water-catchment categories–watersheds separated by Level 1 stream–was significant (*P* = 0.044) before, but not after Bonferroni correction for multiple comparisons.

### Landscape effects on connectivity

Although *F*_*ST*_-values between sites within the same region were highly variable (and were sometimes of similar magnitude to *F*_*ST*_-values between regions), our analysis did not identify a significant predictor of this differentiation. Of the five predictor variables, the best fit model included only one predictor–the proportion of shrub/scrub area between sampled locations (*P* = 0.055).

### Influence of disturbance events on genetic diversity

Both types of disturbance–fire and logging–were common in the study area. Eleven sites had a recent history of fire, and eight sites had a recent history of logging. Five sites had been both burned and logged, and only one logged site had forested buffers left along the waterway.

Evidence of recent bottlenecks was apparent in four sampled sites: sites 2, 12, 20, and 23 (*P* = 0.031, 0.047, 0.016, and 0.016, respectively; S3 Table). However, according to the metrics we considered, only half (two) of these bottlenecked sites had a recent (≤ 70 years) history of disturbance–one had evidence of both fire and logging, and the other only had evidence of fire.

There was also no detected relationship between average allelic richness and recent disturbance events linked to logging and fires, or regarding the preservation of riparian buffers along streams (all *P*-values for Kruskal-Wallis tests > 0.1; S2 Fig).

## Discussion

Species with limited movements that are sensitive to environmental changes and occupy disturbance-prone habitats may experience greater population genetic variation. These genetic signatures may reflect the consequences of historical events, such as colonization of new areas in response to habitat changes from glacial retreat, in conjunction with responses from contemporary environmental changes. In combination, historical and contemporary effects on genetic diversity may compound the effects of future hazards. Our analyses of coastal giant salamanders showed evidence of lower genetic diversity among northern regions (Fig 2) that is likely the legacy of historical disturbance associated with Pleistocene climate change. In addition, we found evidence of moderate-to-high inbreeding and losses of heterozygosity in local populations throughout the study area, despite apparent within-region connectivity and a lack of strong evidence for genetic bottlenecks.

Although impacts on genetic variation are more readily apparent from historical versus recent disturbances, this does not indicate hardiness to contemporary disturbance. It may be that: 1) sensitivity in this species is more context-dependent; 2) there has not been sufficient time for disturbances to leave a genetic signature; 3) signals of historical disturbance have overpowered those of more recent disturbances; 4) our results are statistical false negatives; or 5) some combination of these. Indeed, the first reason is perhaps the primary driver for the lack of a strong detected effect of a particular recent disturbance regime (e.g., logging and fire frequency; S2 Fig). Other types of recent disturbances could not be adequately evaluated, such as debris-flow events in montane headwaters. While studies of this species have shown individual mobility and abundance can be detrimentally affected by logging [34, 43, 85, 86], populations may experience upticks after logging (before the presumed onset of population declines) [87]. This is speculated to be a temporary effect of increased solar input, which increases stream productivity for a time [87]. Additionally, populations of coastal giant salamanders occupying steeper streams have been shown to be less affected by logging [85, 87], presumably because there is less associated siltation. However, logging of steeper terrain is also more likely to result in longer-term ecological problems, such as increased erosion, which would likely negatively impact both headwater and lower elevation habitats. Thus, the unclear genetic consequences of anthropogenic change in our data are perhaps unsurprising, and, in conjunction with the lower genetic diversity in the northern region of our study site given historical restrictions in gene flow, potentially warrant future conservation concern. In addition, the species’ relatively long generation time (10–15 years; [88]) may contribute to the lag in genetic signatures.

### Indicators regarding population stability

In our study, local genetic differentiation of coastal giant salamanders (within regional clusters; Fig 4B) was sometimes as high as differentiation of sites between regions (Fig 4A). However, this local genetic differentiation does not correspond with geographic distance, disturbance factor (e.g., fire and logging records), or drainage membership (Fig 4B). Many explanations might apply to any one of these observations, but there is no obvious overarching explanation when these three factors (distance, disturbance, drainage membership) are considered together. Especially given that none of the *F*_*ST*_-values are high in an absolute sense, the genetic differences within regional clusters may or may not reflect the deterministic effects of unmeasured environmental factors or genetic drift.

Consideration of additional results–namely, lack of strong evidence for population bottlenecks (S3 Table), but moderate levels of inbreeding and loss of heterozygosity (S1 and S3 Tables)–would seem at odds with the moderate levels of gene flow suggested by the overall low *F*_*ST*_-values (Fig 4). Any explanation for patterns of genetic differentiation within regions (i.e., connectivity as determined by low *F*_*ST*_-values, but highly variable *F*_*ST*_-values among sites within regions, and a lack of correspondence with fire or logging regimes) must also be compatible with the lack of strong evidence for population bottlenecks, moderate levels of inbreeding, and loss of heterozygosity (i.e., lower observed levels of heterozygosity than expected; S1 and S3 Tables).

When these findings are considered jointly, they are consistent with a general lack of population persistence, and specifically, locally fluctuating population sizes. Such fluctuations would drive down the effective population sizes, which could explain the inbreeding and loss of heterozygosity. Fluctuating population sizes, in particular, could also lead to the variation in *F*_*ST*_-values observed within regions (i.e., these values are sometimes very low, but other times higher than those of inter-regional sites; as seen by comparing the spread of the black versus gray dots in Fig 4). Variation in *F*_*ST*_-values did not correspond with disturbance factors (i.e., any statistical association may be masked by fluctuations; S3 Table). Irrespective of the debate that might be leveled at this specific interpretation, dispersal among sites, as indicated by collectively low *F*_*ST*_-values, may not be sufficient to compensate demographically for potential population vulnerability arising from inbreeding and loss of heterozygosity. The temporal mosaic of disturbance (even within contemporary environmental changes), expounded upon above, might contribute to unclear, mixed genetic signals.

### Implications for conservation under historical and contemporary disturbance

Whereas other studies of PNW salamanders have shown that genetic structure is affected by habitat disturbance associated with logging (e.g., coastal giant salamanders) [34] or land-cover type and roads (e.g., *Rhyacotriton* spp.) [45], we found genetic differentiation was not explained by any factors we tested (type of disturbance events, land cover type, distance, or watersheds/drainages; S3 Table). We caution that the lack of a significant predictor pertains only to our dataset (e.g., with denser local sampling, such effects might emerge) [45]. The difference between this study and previous ones are not necessarily contradictory. Our work incorporates both regional and local components not found in past studies. Moreover, our sampling spans a range of heterogeneous conditions within a region to test for more “global” explanatory factors for local genetic structure (i.e., shared effects across each individual region, such as the importance of drainage or watershed membership). Past studies have suggested that the rarity of *Rhyacotriton* spp. in contrast to coastal giant salamanders [40] may indicate differing sensitivities to disturbance.

Similarly, it would be a mistake to make management decisions based on the presumption that gene flow will resolve negative impacts of disturbance (e.g., fire and logging). The sometimes-high genetic differentiation within a region (compared with between regions: Fig 4A) suggests demographic events capable of causing drift-induced genetic differences, inbreeding, and loss of heterozygosity (S1 Table). Moreover, if potential fluctuating population sizes (described above) obscure effects of local landscape factors, our analyses do not speak to the lack of importance of the environment on population connectivity, but only to lack of detectability given the data. Since our focal species breeds in naturally fragmented and disturbance-prone small-stream habitats [22, 26, 27] and transforms to terrestrial forms that inhabit riparian and upland forests, disturbance to any one of the habitats may affect local population persistence (e.g., [22]). Also, it is possible that there may be genetic signatures in these salamanders of past disturbances, such as older fires or debris flows unrelated to recent fire and logging. In any event, our data suggests that because of detected bottlenecks and high inbreeding (*F*_*IS*_) levels, coastal giant salamanders may be vulnerable to genetic losses from unspecified causes.

It is possible that a higher dispersal capacity contributes to the low regional genetic differentiation and a pattern of isolation by distance (although this wouldn’t explain the lower genetic diversity in northern sites; Fig 2, S1 Table). Species reliant upon naturally fragmented habitats and subject to periodic disturbance can have correspondingly high dispersal proclivities that facilitate recolonization, thereby avoiding permanent extirpations [89]. Thus, relatively high dispersal propensities in coastal giant salamanders may be advantageous if habitats are naturally subject to periodic disturbance, as may be the case with headwater habitats due to landslides [90]. High dispersal propensity has also been suggested in other salamander species which similarly rely on ephemeral ponds [91]. However, this explanation alone may be contradicted by the observed genetic differentiation at local geographic scales, which sometimes matches that observed at much larger, regional scales (Fig 4A).

Studies of other amphibians at similar geographic scales will be important for testing the patterns observed here and evaluate our proposed interpretations. Species comparative studies in the context of cold-survival strategies and sensitives could provide guidance on how different amphibians were affected by, and recovered from, past glaciation [92]. Such comparative studies will also determine whether amphibian communities might be subject to the potentially damaging effects of inbreeding and reduced heterozygosity, despite different degrees of genetic structure (i.e., connectivity). If so, this would suggest that viewing genetic structure, or lack thereof, as a measure of greater resilience due to higher gene flow levels may be misleading. While some species might be capable of higher dispersal and recolonization rates, if the local populations are nonetheless subject to fluctuations (resulting in inbreeding and reduced heterozygosity), such species are not necessarily less vulnerable than those with reduced gene flow (which creates a patchwork of small, isolated populations that also experience strong inbreeding and reduced heterozygosity) [93]. Even though the coastal giant salamander has not been identified as a species of conservation concern, they exhibit levels of inbreeding and reduced heterozygosity that are odds with a robust species, possibly placing them in a precarious situation for dealing with future perturbations such as climate change and diseases [46]. For salamanders, and other relatively immobile species potentially still recovering from past glaciation events, management for enhanced connectivity (such as the development of corridors that span political boundaries [94]) may be key, but may not be sufficient as a sole mitigation strategy.

## Supporting information

S1 Text Results excluding locus D6. Summary of main results when locus D6, which is apparently not in HW equilibrium, is excluded.
(PDF)

S1 Table Summary statistics of the five loci used in this study. Loci (far left column) are summarized per sampled site (1–23). The summary statistics described are number of individuals with information on that locus (*n*), observed heterozygosity (*H*_*o*_), expected heterozygosity (*H*_*e*_), expected heterozygosity under Hardy-Weinberg equilibrium (HWE), the number of alleles (NA), the allelic richness (AR), and inbreeding coefficient (*F*_*IS*_).
(PDF)

S2 Table Corresponding hydrology information for sampled sites (1–23). These include corresponding watershed units: (HUs 08, 10, and 12; U.S. Geological Survey, 2001), drainage basin, or watershed subdivided by Level 1 streams (Lev1; Esri, 2010). For HUs, numbers correspond to the unique HU identifier codes designated by the U.S. Geological Survey [72]. Numbers associated with “Drainage” and “Lev1” classifications are arbitrary, but similarly intended to show subdivision membership.
(PDF)

S3 Table Genetic diversity metrics for each population and landscape characteristics. For each site (1–23), mean *H*_*o*_, mean *H*_*e*_, and mean *F*_*IS*_, and the total number of alleles (NA). Estimates for expected heterozygosity given mutation-drift equilibrium (*H*_*eq*_) and whether the populations has likely experienced a genetic bottleneck (*P*-values) are based on the program BOTTLENECK [82]. Dashes represent populations which were not evaluated due to low sample sizes, and asterisks denote populations with significant likelihood of having experienced a bottleneck. Also recorded is whether or not (Y/N; yes/no), in the past 70 years, there was evidence of disturbance (Fire or Logging) and if the forested buffer remained undisturbed (i.e., Y means that yes, the buffer remained intact).
(PDF)

S1 Fig Plot of genetic Structure results. Individuals (vertical bars) are grouped by population and organized according to sampled site (labels in three horizontal rows; see Fig 1 for geographic details). Results are presented for the most likely number of genetic clusters for each scenario (either *K* = 4 or *K* = 3) as shown by the different colors. The three scenarios include *a prior* categorization of individuals (i.e. conditioning) by (i) watershed (based on HU 08 categorization) and (ii) population, as well as with (iii) no *a priori* information for individuals.
(PDF)

S2 Fig Boxplots of allelic richness versus disturbance history. Allelic richness (y-axis) refers to the average allelic richness for each site, and this was compared to whether or not the sites experienced a disturbance event (burning, logging, or having the riparian buffer disturbed by fire or logging) within the last 70 years. Kruskal-Wallis tests (three) did not identify statistically significant effects of these factors on allelic richness.
(PDF)
