# Supplementary material for: Landscape connectivity among coastal giant salamander (Dicamptodon tenebrosus) populations shows no association with land use, fire frequency, or river drainage but exhibits genetic signatures of potential conservation concern
Source: PLoS One. 2022 Jun 8;17(6):e0268882. doi: 10.1371/journal.pone.0268882 (PMC9176808; doi:10.1371/journal.pone.0268882)
Supplement: S1 Text — Summary of main results when locus D6, which is apparently not in HW equilibrium, is excluded. (PDF) [file pone.0268882.s001.pdf]

Results excluding locus D6

Accompanies the publication: Vulnerability of coastal giant salamanders (*Dicamptodon tenebrosus*)? The threats that may obscure effects of land-use, fire frequency, or river drainage effects on genetic variation

Because locus D6 did not pass the test for Hardy Weinberg Equilibrium, results without the locus are presented here. These results do not differ substantially from those presented in the main manuscript. Pairwise F_{ST} between sites ranged from 0.0 to 0.28. The PCA and STRUCTURE [60] results did not differ substantially, with the former still showing reduced genetic in northern populations and the latter still supporting $K = 4$, generally divided by region. Similarly, IBD was still significant across regions ($P \approx 0.002$), but not within regions ($P = 0.888$), and water catchments were not significantly related to IBD. When residuals were considered in the context of the five different watershed delimitation methods, watersheds separated by category 1 (large) streams was still significant ($P = 0.022$) before, but not after Bonferroni correction for multiple comparisons. Another watershed classification (drainage basin) was significant, but the trend was in the opposite direction we would expect if different drainages led to higher-than-expected F_{ST} between sites (both residuals and F_{ST} tended to be higher if sites were sampled in the same Drainage River match).
