# Supplementary material for: Landscape connectivity among coastal giant salamander (Dicamptodon tenebrosus) populations shows no association with land use, fire frequency, or river drainage but exhibits genetic signatures of potential conservation concern
Source: PLoS One. 2022 Jun 8;17(6):e0268882. doi: 10.1371/journal.pone.0268882 (PMC9176808; doi:10.1371/journal.pone.0268882)
Supplement: S1 Table — Loci (far left column) are summarized per sampled site (1–23). The summary statistics described are number of individuals with information on that locus (n), observed heterozygosity (Ho), expected heterozygosity (He), expected heterozygosity under Hardy-Weinberg equilibrium (HWE), the number of alleles (NA), the allelic richness (AR), and inbreeding coefficient (FIS). (PDF) [file pone.0268882.s002.pdf]

		**Region A**						**Region B**					**Region C**						**Region D**		**Region E**			
Loc		**1**	**2**	**3**	**4**	**5**	**6**	**7**	**8**	**9**	**10**	**11**	**12**	**13**	**14**	**15**	**16**	**17**	**18**	**19**	**20**	**21**	**22**	**23**
D6	n	8	18	5	13	6	4	10	17	19	18	11	11	17	11	11	25	29	13	6	14	12	15	15
	H_o	0.13	0.00	0.00	0.15	0.17	0.00	0.40	0.41	0.32	0.39	0.36	0.36	0.41	0.36	0.55	0.44	0.21	0.62	0.50	0.50	0.33	0.93	0.53
	H_e	0.86	0.88	0.90	0.88	0.87	0.83	0.81	0.80	0.78	0.91	0.90	0.90	0.92	0.84	0.77	0.72	0.64	0.79	0.92	0.89	0.85	0.87	0.85
	HWE	0.75	0.82	0.73	0.81	0.79	0.64	0.73	0.70	0.75	0.88	0.85	0.82	0.89	0.88	0.82	0.74	0.77	0.77	0.89	0.81	0.78	0.83	0.83
	NA	5	8	4	7	5	3	5	5	6	11	8	7	11	9	7	6	7	6	7	7	6	8	8
	AR	3.71	4.27	3.60	4.17	3.81	2.93	3.60	3.64	3.58	4.68	4.42	4.32	4.77	4.09	3.67	3.33	2.87	3.68	4.52	4.33	3.97	4.40	4.13
	F_{IS}	0.85	1.00	1.00	0.82	0.81	1.00	0.50	0.49	0.60	0.57	0.60	0.59	0.55	0.57	0.29	0.39	0.68	0.22	0.46	0.44	0.61	-0.07	0.37
D14	n	8	12	3	13	5	5	9	17	19	19	11	11	17	12	11	27	29	13	6	14	12	15	15
	H_o	0.75	0.92	1.00	0.69	0.60	0.60	0.78	0.77	0.63	0.84	0.82	0.82	0.88	0.92	0.73	0.78	0.90	1.00	0.50	0.64	0.58	0.60	0.73
	H_e	0.96	0.89	0.92	0.93	1.00	0.78	0.93	0.92	0.87	0.91	0.91	0.86	0.88	0.75	0.59	0.83	0.92	0.89	0.85	0.90	0.81	0.73	0.91
	HWE	0.93	0.87	0.93	0.93	0.95	0.82	0.92	0.91	0.87	0.90	0.92	0.85	0.89	0.82	0.78	0.87	0.91	0.92	0.85	0.88	0.72	0.76	0.89
	NA	10	9	5	13	8	5	10	13	10	12	11	8	11	7	6	11	15	12	6	10	5	6	11
	AR	5.16	4.63	5.00	4.95	5.33	3.67	4.94	4.86	4.38	4.77	4.82	4.25	4.56	3.69	2.99	4.05	4.95	4.75	4.04	4.57	3.66	3.42	4.71
	F_{IS}	0.22	-0.03	-0.09	0.26	0.40	0.23	0.16	0.17	0.28	0.07	0.10	0.04	0.00	-0.22	-0.24	0.06	0.03	-0.12	0.41	0.28	0.28	0.18	0.19
D17	n	8	20	5	13	6	5	11	17	19	19	11	10	17	10	10	23	28	12	4	11	10	13	13
	H_o	0.88	0.55	0.20	0.54	0.50	0.60	0.27	0.35	0.32	0.74	0.82	0.40	0.18	0.60	0.50	0.44	0.64	0.25	0.75	0.55	0.40	0.23	0.15
	H_e	0.80	0.80	0.80	0.79	0.58	0.68	0.69	0.82	0.82	0.86	0.87	0.88	0.81	0.86	0.92	0.89	0.85	0.83	0.92	0.93	0.89	0.91	0.87
	HWE	0.75	0.74	0.73	0.77	0.79	0.82	0.73	0.75	0.75	0.84	0.85	0.83	0.79	0.83	0.89	0.84	0.87	0.78	0.86	0.88	0.86	0.84	0.77
	NA	5	6	4	6	5	5	5	6	6	9	8	7	7	7	9	9	11	6	5	9	8	8	6
	AR	3.71	3.72	3.33	3.62	3.00	3.40	3.14	3.72	3.79	4.26	4.40	4.22	3.73	4.18	4.67	4.46	4.20	3.74	4.39	4.76	4.39	4.43	4.04
	F_{IS}	-0.09	0.31	0.75	0.31	0.14	0.11	0.61	0.57	0.61	0.14	0.06	0.55	0.78	0.30	0.46	0.51	0.24	0.70	0.18	0.41	0.55	0.75	0.82
Dte 11	n	8	20	5	13	6	5	11	17	19	19	10	11	17	12	10	28	29	13	6	13	12	15	14
	H_o	0.13	0.75	0.40	0.54	0.67	0.60	Monomorphic	0.41	0.11	0.53	0.10	0.36	0.65	0.17	0.10	0.04	0.35	Monomorphic	Monomorphic	0.62	0.67	0.40	0.50
	H_e	0.34	0.67	0.55	0.58	0.68	0.60		0.47	0.20	0.54	0.10	0.31	0.45	0.40	0.10	0.07	0.38			0.71	0.76	0.57	0.82
	HWE	0.33	0.48	0.38	0.51	0.58	0.60		0.49	0.48	0.61	0.31	0.31	0.28	0.30	0.31	0.47	0.46			0.63	0.72	0.62	0.71
	NA	2	3	2	3	3	3		3	3	4	2	2	2	2	2	3	3			4	5	4	5
	AR	1.79	2.75	2.00	2.48	2.76	2.57		2.29	1.57	2.61	1.30	1.75	1.93	1.86	1.30	1.21	2.05			3.05	3.47	2.35	3.76
	F_{IS}	0.63	-0.12	0.27	0.08	0.02	0.00		0.13	0.47	0.03	0.00	-0.18	-0.46	0.59	0.00	0.50	0.09			0.13	0.12	0.29	0.39
Dte 14	n	8	18	5	13	6	6	11	17	19	19	11	10	17	12	5	18	18	13	6	13	6	15	10
	H_o	0.00	0.11	0.40	0.00	0.00	0.17	0.00	0.18	0.05	0.42	0.82	0.40	0.18	0.17	0.00	0.33	0.17	0.39	1.00	0.46	0.17	0.47	0.30
	H_e	0.25	0.46	0.38	0.28	0.53	0.57	0.47	0.49	0.60	0.72	0.59	0.65	0.41	0.71	0.70	0.54	0.50	0.77	0.75	0.66	0.47	0.61	0.71
	HWE	0.33	0.49	0.60	0.29	0.36	0.36	0.52	0.49	0.49	0.61	0.52	0.53	0.61	0.52	0.60	0.49	0.49	0.71	0.71	0.51	0.58	0.50	0.65
	NA	2	3	3	2	2	2	3	3	3	4	3	3	4	3	3	3	3	5	4	3	3	3	4
	AR	1.63	2.05	2.20	1.68	1.97	1.99	2.23	2.19	2.38	3.06	2.45	2.63	2.15	2.78	2.73	2.14	2.10	3.39	3.35	2.71	2.27	2.60	2.93
	F_{IS}	1.00	0.76	-0.07	1.00	1.00	0.71	1.00	0.64	0.91	0.41	-0.39	0.39	0.57	0.76	1.00	0.39	0.67	0.50	-0.33	0.30	0.64	0.23	0.58
