# Supplementary material for: Landscape connectivity among coastal giant salamander (Dicamptodon tenebrosus) populations shows no association with land use, fire frequency, or river drainage but exhibits genetic signatures of potential conservation concern
Source: PLoS One. 2022 Jun 8;17(6):e0268882. doi: 10.1371/journal.pone.0268882 (PMC9176808; doi:10.1371/journal.pone.0268882)
Supplement: S2 Table — These include corresponding watershed units: (HUs 08, 10, and 12; U.S. Geological Survey, 2001), drainage basin, or watershed subdivided by Level 1 streams (Lev1; Esri, 2010). For HUs, numbers correspond to the unique HU identifier codes designated by the U.S. Geological Survey [72]. Numbers associated with “Drainage” and “Lev1” classifications are arbitrary, but similarly intended to show subdivision membership. (PDF) [file pone.0268882.s003.pdf]

| Site | HU08 | HU10 | HU12 | Drainage | Lev1 |
|---|---|---|---|---|---|
| 1 | 238 | 2097 | 9113 | 2 | 1 |
| 2 | 238 | 2097 | 9113 | 2 | 1 |
| 3 | 238 | 2097 | 9113 | 2 | 1 |
| 4 | 238 | 2097 | 9115 | 2 | 2 |
| 5 | 238 | 2097 | 9115 | 2 | 2 |
| 6 | 238 | 2097 | 9114 | 2 | 1 |
| 7 | 195 | 1837 | 11067 | 3 | 3 |
| 8 | 195 | 1837 | 11067 | 3 | 3 |
| 9 | 240 | 2101 | 9176 | 4 | 4 |
| 10 | 195 | 1837 | 11065 | 3 | 3 |
| 11 | 238 | 1817 | 8416 | 2 | 5 |
| 12 | 240 | 2107 | 9185 | 1 | 6 |
| 13 | 240 | 2107 | 9185 | 4 | 7 |
| 14 | 240 | 2107 | 9185 | 4 | 8 |
| 15 | 240 | 2107 | 9185 | 4 | 9 |
| 16 | 240 | 2107 | 9185 | 4 | 10 |
| 17 | 240 | 2107 | 9185 | 1 | 11 |
| 18 | 241 | 2121 | 9074 | 5 | 12 |
| 19 | 179 | 2046 | 10196 | 6 | 13 |
| 20 | 180 | 2049 | 11268 | 7 | 14 |
| 21 | 181 | 2060 | 10635 | 8 | 15 |
| 22 | 181 | 2060 | 10635 | 8 | 15 |
| 23 | 180 | 2049 | 11266 | 7 | 16 |
