# Supplementary material for: Landscape connectivity among coastal giant salamander (Dicamptodon tenebrosus) populations shows no association with land use, fire frequency, or river drainage but exhibits genetic signatures of potential conservation concern
Source: PLoS One. 2022 Jun 8;17(6):e0268882. doi: 10.1371/journal.pone.0268882 (PMC9176808; doi:10.1371/journal.pone.0268882)
Supplement: S3 Table — For each site (1–23), mean Ho, mean He, and mean FIS, and the total number of alleles (NA). Estimates for expected heterozygosity given mutation-drift equilibrium (Heq) and whether the populations has likely experienced a genetic bottleneck (P-values) are based on the program BOTTLENECK [82]. Dashes represent populations which were not evaluated due to low sample sizes, and asterisks denote populations with significant likelihood of having experienced a bottleneck. Also recorded is whether or not (Y/N; yes/no), in the past 70 years, there was evidence of disturbance (Fire or Logging) and if the forested buffer remained undisturbed (i.e., Y means that yes, the buffer remained intact). (PDF) [file pone.0268882.s004.pdf]

Region	Site	H_o	H_e	H_{eq}	*P*	F_{IS}	NA	Fire	Logging	Buffer
**A**	**1**	0.38	0.62	—	—	0.42	24	N	Y	Y
	**2**	0.47	0.73	0.68	0.03*	0.37	29	N	N	Y
	**3**	0.4	0.68	—	—	0.44	18	N	N	Y
	**4**	0.38	0.68	0.66	0.31	0.44	31	N	Y	N
	**5**	0.39	0.7	—	—	0.47	23	N	N	Y
	**6**	0.39	0.65	—	—	0.43	18	N	Y	N
**B**	**7**	0.29	0.57	—	—	0.5	23	N	N	Y
	**8**	0.42	0.69	0.67	0.11	0.4	30	N	N	Y
	**9**	0.28	0.64	0.67	0.31	0.57	28	N	N	N
	**10**	0.58	0.78	0.77	0.11	0.26	40	Y	N	N
	**11**	0.58	0.67	0.69	0.41	0.14	32	N	N	Y
**C**	**12**	0.47	0.71	0.67	0.05*	0.35	27	Y	Y	N
	**13**	0.46	0.69	0.69	0.59	0.34	35	Y	N	N
	**14**	0.44	0.7	0.67	0.59	0.38	28	Y	N	N
	**15**	0.37	0.6	—	—	0.39	27	Y	N	N
	**16**	0.4	0.61	0.68	0.89	0.34	32	Y	Y	N
	**17**	0.45	0.65	0.7	0.92	0.31	39	Y	Y	N
**D**	**18**	0.45	0.65	—	—	0.31	29	Y	Y	N
	**19**	0.55	0.67	—	—	0.2	22	N	N	Y
**E**	**20**	0.55	0.8	0.74	0.02*	0.32	33	Y	N	N
	**21**	0.43	0.74	—	—	0.43	27	Y	Y	N
	**22**	0.53	0.73	0.71	0.08	0.29	29	Y	N	N
	**23**	0.44	0.82	0.77	0.02*	0.47	34	N	N	Y
