# Supplementary figures and images for: Landscape connectivity among coastal giant salamander (Dicamptodon tenebrosus) populations shows no association with land use, fire frequency, or river drainage but exhibits genetic signatures of potential conservation concern
Source: PLoS One. 2022 Jun 8;17(6):e0268882. doi: 10.1371/journal.pone.0268882 (PMC9176808; doi:10.1371/journal.pone.0268882)

Conditioning  
by drainage  
 $K = 4$

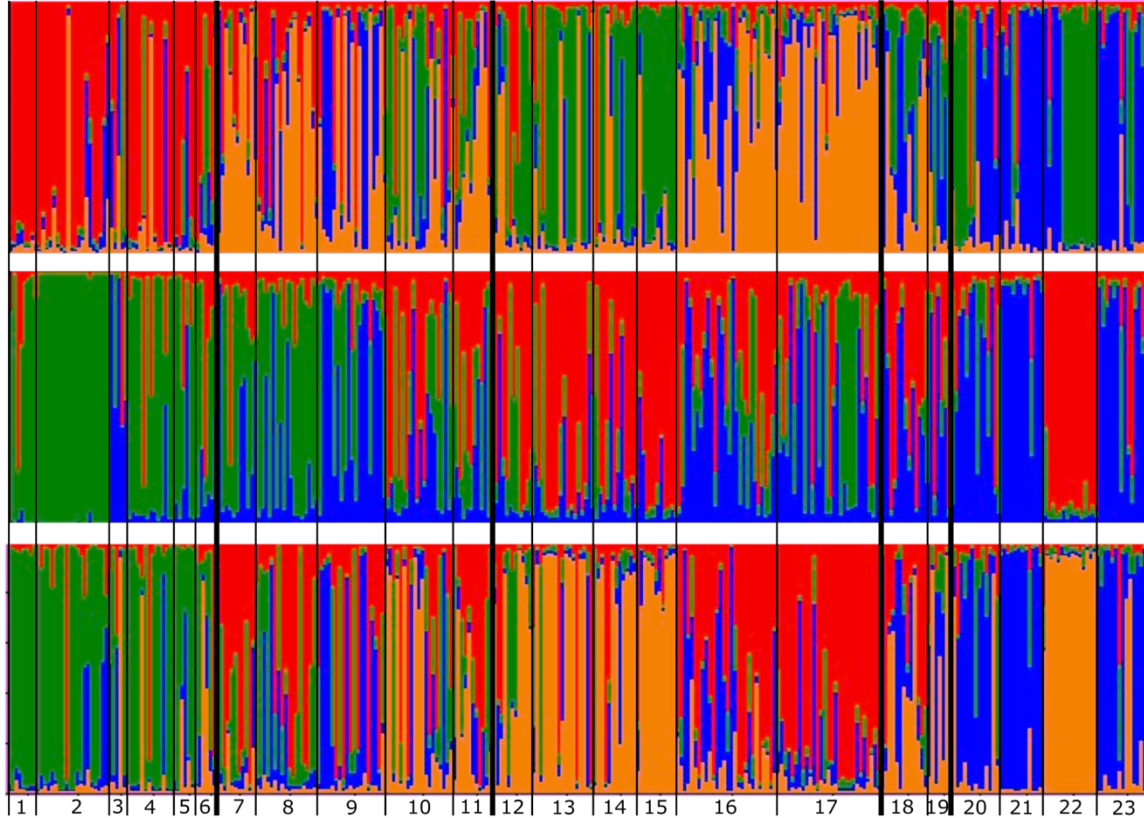

Supplement: S1 Fig — Individuals (vertical bars) are grouped by population and organized according to sampled site (labels in three horizontal rows; see Fig 1 for geographic details). Results are presented for the most likely number of genetic clusters for each scenario (either K = 4 or K = 3) as shown by the different colors. The three scenarios include a prior categorization of individuals (i.e. conditioning) by (i) watershed (based on HU 08 categorization) and (ii) population, as well as with (iii) no a priori information for individuals. (PDF) [file pone.0268882.s005.pdf]

Allelic Richness

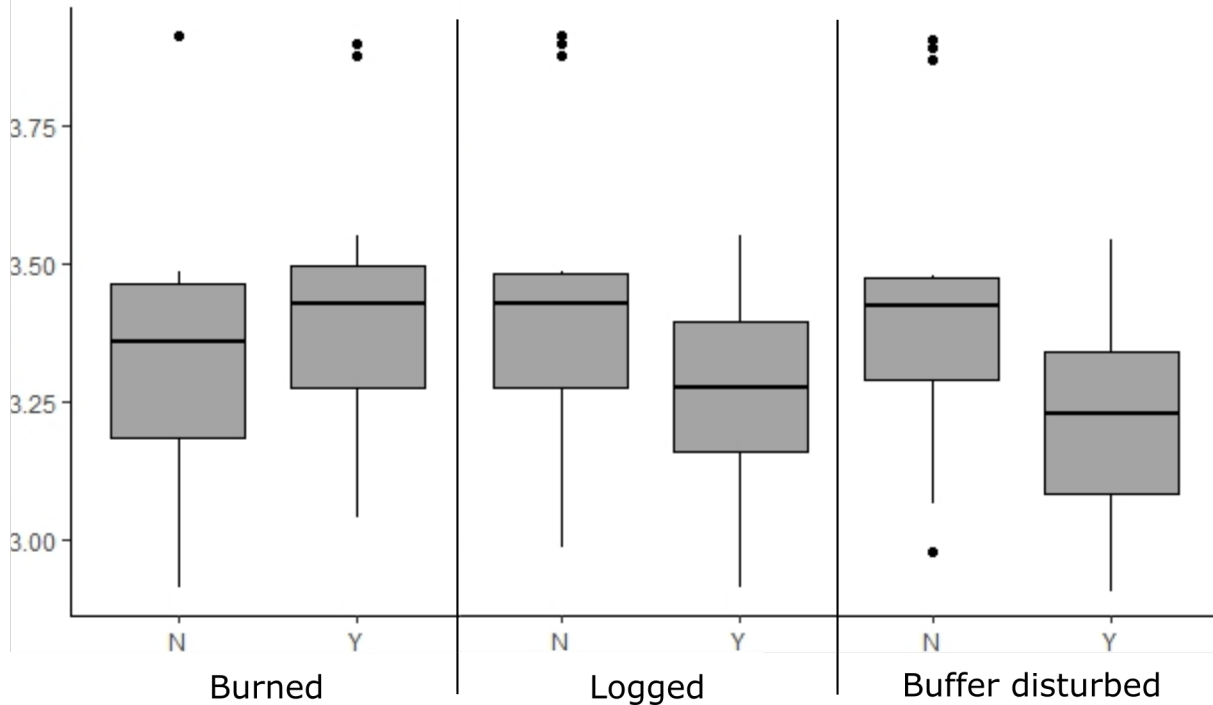

Supplement: S2 Fig — Allelic richness (y-axis) refers to the average allelic richness for each site, and this was compared to whether or not the sites experienced a disturbance event (burning, logging, or having the riparian buffer disturbed by fire or logging) within the last 70 years. Kruskal-Wallis tests (three) did not identify statistically significant effects of these factors on allelic richness. (PDF) [file pone.0268882.s006.pdf]
